# Current state and trends of access to sanitation in Ethiopia and the need to revise indicators to monitor progress in the Post-2015 era

**DOI:** 10.1186/s12889-015-1804-4

**Published:** 2015-05-02

**Authors:** Abebe Beyene, Tamene Hailu, Kebede Faris, Helmut Kloos

**Affiliations:** Department of Environmental Health Science and Technology, Jimma University, P. O. Box: 378, Jimma, Ethiopia; Research and Development Directorate, Ministry of Water, Irrigation & Energy (MoWIE), P. O. Box: 5744, Addis Ababa, Ethiopia; Water & Sanitation Program (WSP), the World Bank, Ethiopia Country Office, P. O. Box: 5515, Addis Ababa, Ethiopia; Department of Epidemiology and Biostatistics, University of California, 185 Berry Street, Box 0560, San Francisco, CA 94143 – 0560 USA

**Keywords:** Improved sanitation coverage, Sanitation trend, Sanitation ladder, Millennium development goals, Ethiopia

## Abstract

**Background:**

Investigating the current level and trends of access and identifying the underlying challenges to sanitation system development will be useful in determining directions developing countries are heading as they plan to promote sustainable development goals (post 2015 agenda). This research investigates the status and trends of access to improved sanitation coverage (ISC) in relation to the MDG target in Ethiopia with the aim of identifying prevailing constraints and suggesting the way forward in the post-MDG era.

**Method:**

We examined data from a nationwide inventory conducted in accordance with the sanitation ladder at the national level and from a household survey in randomly selected urban slums in Addis Ababa. The inventory data were analyzed and interpreted using the conceptual model of the sanitation ladder. We used administrative reports and survey results to plot the time trend of the ISC.

**Results:**

The data from the nationwide inventory of sanitation facilities, which are presented along the sanitation ladder reveal that more than half of the Ethiopian population (52.1%) still used unimproved sanitation facilities in 2014. The majority (35.6%) practiced open defecation, implying that the country is far from the MDG target for access to improved sanitation (56%). Most people in urban slums (88.6%) used unimproved sanitation facilities, indicating that the urban poor did not receive adequate sanitation services. Trend analysis shows that access to ISC has increased, but Central Statistical Authority (CSA) data reveal a decline. This discrepancy is due to differences in data collection methods and tools. Dry pit latrines are the most widely used toilet facilities in Ethiopia, accounting for about 97.5% of the ISC.

**Conclusion:**

The sanitation coverage is far from the MDG target and the majority of the population, mainly the urban poor, are living in a polluted environment, exposed to water and sanitation-related diseases. The sanitation coverage estimates might be even lower if proper utilization, regular emptying, and fecal sludge management (FSM) of dry pit latrines were considered as indicators. In order to enhance sanitation services for all in the post-MDG era, urgent action is required that will establish proper monitoring and evaluation systems that can measure real access to ISC.

## Background

Lack of access to sanitation, use of unsafe drinking water, and poor hygiene together are responsible for about 88% of all deaths from diarrheal diseases in developing countries [1]. Sanitation and health experts are also estimated that improved sanitation alone could reduce by one third the global incidence of diarrheal disease, a leading killer of children [2], and can also play a major role in reducing parasitic infections that impede child development. Cognizant of the crucial role of water, sanitation, and hygiene in health development, the United Nations (UN), in Resolution 64/292, explicitly recognizes the human right to water and sanitation [3]. This resolution declares that safe drinking water and sanitation are essential to the realization of all human rights and calls upon states and organizations to support developing countries in the provision of safe, adequate, and accessible drinking water and sanitation for all. In support of this UN resolution, the World Health Organization (WHO), in Resolution 64/24, urges member states to ensure that national health strategies contribute to the realization of water- and sanitation-related Millennium Development Goals (MDGs) [4].

An improved sanitation facility is commonly defined as one that hygienically separates human excreta from human contact [5]. According to the Joint Monitoring Program for Water Supply and Sanitation (JMP) [5], improved sanitation coverage (ISC) is measured as the proportion of a population using an improved sanitation facility. Private improved pit latrines (PIPL), private traditional pit latrines (PTPL) with slab and super structure, composting toilets, and flush or pour-flush toilets connected to sewer systems and septic tanks are considered improved sanitation (IS); improved shared latrines (ISL), unsanitary toilets (USTs) such as flush or pour-flush toilets that discharge their contents into the environment, pit latrines without super structure, open pit, bucket, hanging toilets, and open defecation (OD) are considered unimproved sanitation (UIS).

Globally, remarkable achievements have been made in the provision of sanitation, with over 64% of the world’s population reportedly having access to improved sanitation in 2013 [6]. In 2014, the WHO and UNICEF JMP reported that 116 countries met the MDG target for drinking water whereas only 64 countries met the target for sanitation. Thirty-seven of the 69 countries not on track to meet the MDG sanitation target were in Sub-Saharan Africa [6].

According to the JMP and the Central Statistical Authority of Ethiopia (CSA) reports, Ethiopia is one of the Sub-Saharan African countries not on track to meet the MDG sanitation target [6,7], although the national report of the Ministry of Finance and Economic Development [8] claims that Ethiopia is on track to meet this MDG target. The discrepancy between these reports may impair progress in improved sanitation coverage because overestimated coverage can result in a false sense of achievement. The rapidly increasing demand for sanitation [9] and the deteriorating rate of access to improved sanitation in Sub-Saharan Africa [10,11], where Ethiopia is a case in point, call for detailed research. Identifying current levels and trends of access and identifying the driving factors will become increasingly important as populations grow larger and struggle to obtain basic services. Therefore, one major objective of this study is to assess the status, trends, and reporting of sanitation in Ethiopia.

In 2010, only 40% of the global population (2.8 billion people) used improved sanitation as estimated by Baum et al. [12]; this figure is little over half the JMP estimate (4.3 billion people) for that year [5]. Baum et al. [12] also estimated that 4.1 billion people globally lacked access to improved sanitation facilities. The discrepancy is due to the inclusion of unimproved sanitation, such as toilet facilities connected to sewer systems without adequate sewage treatment, in the improved sanitation category in the JMP report [12]. Some studies report that sanitation coverage is overestimated due to the use of wrong indicators for improved sanitation [13] and because of over reporting [14]. Monitoring progress in sanitation access has mainly focused on household-level inventories of type and number of toilet facilities, ignoring proper utilization and user behavior [15]. Evaluation of access to improved sanitation should consider the complete fecal sludge management (FSM) chain from containment to adequate treatment, including waste valorization for sustainable sanitation systems. In this regard, detailed studies are required to identify the limitations of the monitoring system and the use of indicators to comprehensively assess sanitation services in relation to their suitability for pollution control and minimizing public health risks. The second objective of this research is therefore to investigate methods and tools useful in increasing accessibility to improved sanitation in Ethiopia, particularly indicators used to monitor progress towards greater access.

## Methods

### Review of reports

The JMP of WHO and UNICEF reports on progress in improved sanitation coverage (ISC) at http://www.wssinfo.org/documents/. We accessed and collected the data points of the JMP reports from this online source for 1990–2014. We also compiled data on ISC trends from administrative government reports (AGRs) of the Ethiopian Ministry of Health (MoH) that are available in its annual Health and Health Related Indicators reports as well as the Ethiopian Demographic Health Survey (DHS) data for 1990–2014. The time trends in these MoH reports were plotted using line charts without smoothing technique to show the real variability within the reports. We critically appraised sanitation survey methods (access and actual use) and use of indicators as well as the system and chain of reporting within the government structure.

### National sanitation inventory

A cross-sectional study design was used in all the surveys. The sanitation ladder used by the JMP [16] is a useful tool for monitoring progress towards MDG 7. In 2014, The Ethiopian Ministry of Water, Irrigation and Energy conducted a nationwide inventory of sanitation facilities in accordance with the sanitation ladder. The inventory was carried out in all urban and rural households nationwide by trained data collectors using an observational checklist and predefined lists of improved and unimproved sanitation facilities [5]. The national representative inventory data were compiled and analyzed to assess the 2014 level of improved sanitation coverage in relation to the MDG target.

### Household survey

To investigate the status of ISC in the poor segment of the population, our study team also conducted a 2014 inventory of sanitation facilities in accordance with the sanitation ladder; the study was conducted in 403 randomly selected households in urban slums in Addis Ababa. The sample size was estimated using the maximum sample size formula. A multistage sampling technique was used to randomly select five subcities from among Addis Ababa’s 10 subcities, 2 districts from each subcity, and 40 households from each district. Only households with per capita income of less than 1.25 US$ per day were included. We briefly explained the purpose of the interview to the respondents and obtained verbal consent at the beginning of each household interview and direct observation of sanitation facilities, giving households the option of declining to participate without repercussions. One adult household member was interviewed in each selected household. Householders absent at the time of the interviews or who refused to be interviewed were deleted from the list and replaced with the nearest household. All surveys were based on households, but access to improved sanitation was expressed in percent of the population by multiplying the number of households by average family size.

Data quality was ensured by training data collectors (environmental health professionals), maintaining strict supervision of research team members, using a standard checklist during direct observation, and practicing double data entry. The questionnaires were translated into the local language and pretested outside the study area.

### Analysis

**Figure 1:**
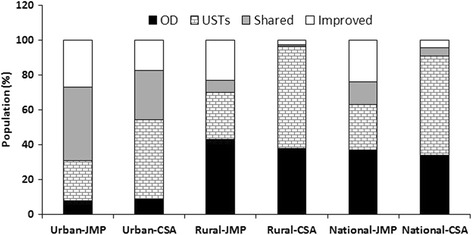
Percentage of the Ethiopian population on the sanitation ladders in urban, rural and national levels. Sources: JMP **=** WHO& UNICEF (2014) and CSA (2014). Note: UST = Unsanitary toilet and OD = Open defecation.

**Figure 2:**
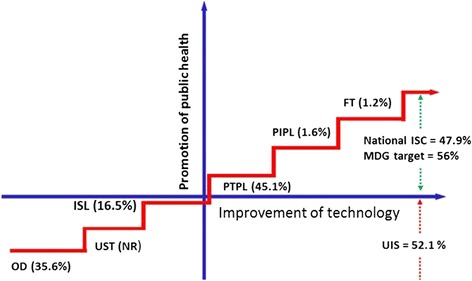
Sanitation ladder adapted from WHO and UNICEF (2008) and national percent sanitation coverage in relation to MDG target. FT = Flush toilet; OD = Open defecation; ISC = Improved sanitation coverage; ISL = Improved shared latrine; NR = Not reported; PIPL = Private improved pit latrine; PTPL = Private traditional pit latrine; UIS = Unimproved sanitation; UST = Unsanitary toilet

Inventory results were analyzed and interpreted using the conceptual model of the sanitation ladder (Figures 1 and 2) adapted from WHO and UNICEF [16]. The adapted sanitation ladder shows sanitation data for Addis Ababa along two axes. The first axis represents the ladders of sanitation technologies from open defecation (OD) to flush toilets (FT) connected to a sewer system or septic tank. The second axis represents the promotion of public health toward access and utilization of improved sanitation facilities that can be measured in terms of the reduction in incidence and prevalence of sanitation-related diseases [16]. Results of this semi-quantitative study were presented in tables and graphs.

### Ethics

The national sanitation inventory was not subjected to ethical review since it is an operational study without involving human subjects. Nevertheless, the protocols for the household survey were reviewed by the Ethical Review Board of the College of Health Sciences, Jimma University, Ethiopia and we received an ethical approval before conducting the survey.

## Results

### Current status of sanitation coverage in relation to the sanitation ladder

We summarized the 2014 sanitation coverage status in Addis Ababa and Ethiopia along the sanitation ladder in Table 1. Only 11.4% of Addis Ababa’s population in the urban slums and 41.2% of the city’s total population had access to improved sanitation. Most people in the urban slums (80.4%) used unimproved sanitation facilities and 8.2% practiced open defecation. Better sanitation and toilet coverage in the urban area of Addis Ababa than in the Addis Ababa slums and national urban areas was indicated by the lower open defecation rate and the generally higher improved sanitation rates in the former (Table 1). According to the 2014 JMP report, 73% of Ethiopia’s urban and 77% of its rural population used unimproved sanitation facilities, with 8% in urban and 43% in rural communities practicing open defecation (Figure 1). The Ethiopian DHS survey in 2014 estimated that 82.5% of the urban and 97.5% of the rural population had no access to improved sanitation and that 8.7% of urban and 37.5% of the rural population practiced open defecation (Figure 1). The use of shared latrines was less common in rural than in urban areas; however, the accessibility rates for unsanitary toilets (USTs) were similar in urban and rural areas (Figure 1).

**Table 1:** **Sanitation coverage at different levels of the sanitation ladder in Addis Ababa and at the national level in 2014**

**Sanitation coverage**	**Sanitation ladder**	**Addis Ababa (% population)**		**National (% population)**		
		**Urban Slum** ^*^	**Urban** ^**^	**Urban** ^***^	**Rural** ^***^	**National** ^** (***)^
**Improved sanitation**	Pour/flush toilet	1.0	20.2	5.3	0.1	1.2 (0.8)
	IPL private	5.2	10.4	0.6	0.1	1.6 (0.2)
	Pit latrine private	5.2	10.6	11.6	2.3	45.1 (3.5)
	Total	11.4	41.2	17.5	2.5	47.9 (4.5)
**Unimproved sanitation**	Shared latrine	58.1	53.0	28.0	1.0	16.5 (4.5)
	UST	22.3	NR	45.8	58.6	NR (56.9)
	Open defecation	8.2	5.8	8.7	37.9	35.6 (34.1)
	Total	88.6	58.8	82.5	97.5	52.1 (95.5)

Note: IPL = improved pit latrine; NR = not reported; UST = unsanitary toilet; * = sample survey; ** = national inventory; *** = CSA (2014).

Extrapolation and comparison of the data of the nationwide inventory of sanitation facilities using the conceptual model of the sanitation ladder shows that 52.1% of Ethiopia’s population use unimproved sanitation facilities and 47.9% have access to improved sanitation facilities. Dry pit latrines (improved pit latrines and pit latrines) are the most common and widely used toilet facilities in Ethiopia (Table 1 and Figure 2). Unsanitary toilets (USTs) such as bucket toilets, open pit toilets, and night soil were considered as open defecation in the national inventory and hence were not included in Figure 2. Of the 52.1% using unimproved sanitation facilities, 35.6% practice open defecation (Figure 2), indicating that Ethiopia is far from the MDG target (56%) for access to ISC.

### Trends of improved sanitation coverage

**Figure 3:**
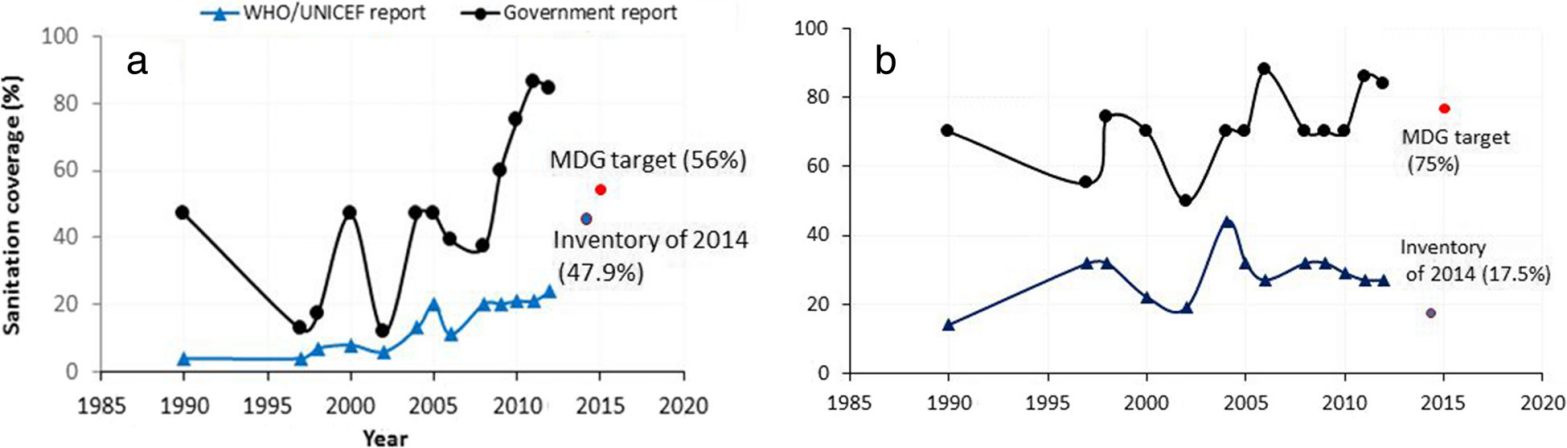
Trends of improved sanitation coverage. **a)** National and **b)** Urban administrative reports of the government compared with the reports of Joint Monitoring Program (JMP) of WHO and UNICEF and the 2014 national sanitation inventory.

A steeper increase of ISC was observed in the AGR than the JMP report on both national (Figure 3a) and urban (Figure 3b) scales. Despite a few discrepancies, sanitation coverage had increased between 1990 and 2012 in both urban areas and nationwide. According to the AGR, ISC increased from 13% in 1997 to 84.1% in 2012 at the national level (Figure 3a), whereas the JMP reported an increase from 4% in 1990 to 24% in 2012 at the national level. The 2014 national level inventory revealed the status of ISC to be 47.9%, which is approximately half way above and half way below the levels reported by the JMP and AGRs, respectively. Similarly, the AGRs showed that ISC increased from 55.0% in 1997 to 83.9% in 2012, whereas JMP reports indicated that ISC increased from 14% in 1990 to 27% in 2012 among urban residents. In contrast to the JMP report, the AGR stated that Ethiopia met the MDG target for access to improved sanitation in 2009. The inventory results estimated that the 2014 status of ISC for urban Ethiopia was lower than 75%. As shown in Figure 3, higher inter-annual variability in the ISC pattern was observed in the AGRs than in the JMP reports.

**Figure 4:**
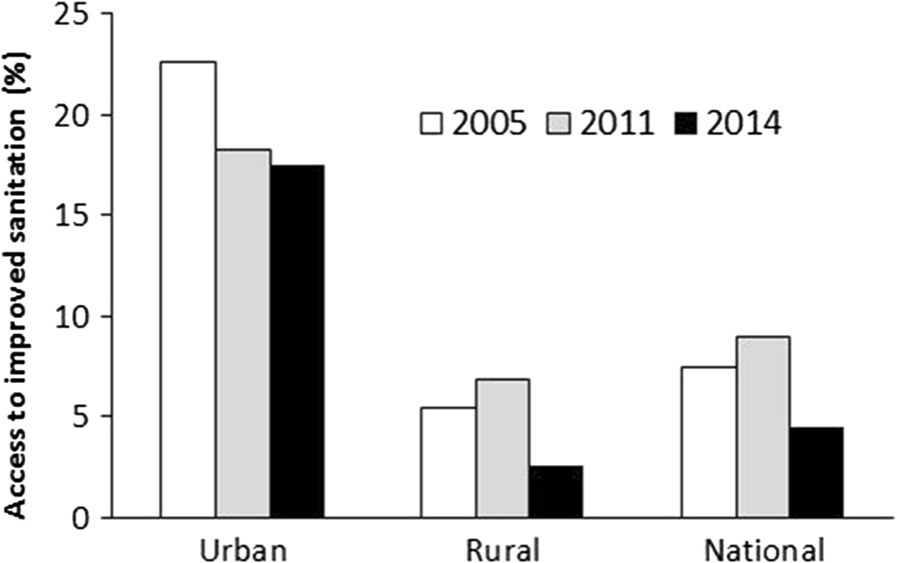
The percentage of the Ethiopian population with access to improved sanitation at the urban, rural and national levels in 2005, 2011 and 2014. Sources: CSA (2014); CSA and CFI International (2012) and CSA and ORC Macro (2006).

In contrast to the AGRs and JMP reports, the results of the national surveys that were conducted by the Ethiopian Statistical Authority in collaboration with international consultants (ORC Macro and CFI International) revealed a declining trend of ISC. For instance, in urban areas, ISC declined from 23.6% in 2005 to 18.2% and 17.5% in 2011 and 2014, respectively (Figure 4) and in rural areas from 6.8% in 2005 to 5.4% in 2011. A two-fold decline in coverage was observed in 2014 at both the rural and national levels compared with 2005 and 2011 (Figure 4).

## Discussion

### Current status of sanitation coverage in relation to the sanitation ladder

The importance of sanitation in safeguarding human health is well known and undisputed. We used the sanitation ladder to analyze the 2014 inventory of sanitation technologies for Ethiopia. This analysis shows that 52.1% of the Ethiopian population still use unimproved sanitation facilities; most practice open defecation. These data indicate that the country is far from the MDG target. The AGRs show that Ethiopia met the interim 2009 MDG target for access to improved sanitation with coverage of 84.1%. The Ministry of Finance and Economic Development (MoFED) reports agree with the AGR, saying that Ethiopia is on track to meet the MDG and showing that the national sanitation coverage increased from 63% in 2010 to 67% in 2012 [8]. Nevertheless, Ethiopian-MoFED stressed in its report that realities on the ground suggest that the country needs to do a lot more to increase access to improved sanitation. In contrast to both the AGR and MoFED reports, the Ethiopian CSA national survey, JMP reports, and the national inventory results confirm that the current state of sanitation is far from the MDG target. Bostoen and Evans [13] pointed out that reports of sanitation coverage for the MDG in most developing countries are unreliable and tend to present an unrealistic sense of achievement. This fact implies that there is a need to improve monitoring tools and the reporting system to minimize discrepancies and facilitate program planning and evaluation.

Due to rapid urbanization and the correspondingly increasing demand for basic sanitation, the claim that urban sanitation in Sub-Saharan Africa has been steadily improving in recent decades is doubtful [17]. Our comparison of the sanitation coverage survey in the urban slums of Addis Ababa with the nationwide sanitation inventory reveals that only 11.4% of urban slum residents have access to improved sanitation. This level of coverage is far lower than the improved sanitation coverage throughout Addis Ababa (41.2%) and the national urban sanitation coverage (27%). Access to ISC in rural areas of the country is only 2.5%. However, access to unsanitary toilets substantially increased, up to 63% in 2014, due to the implementation of the national health extension program by the federal MoH in 2004; the program deployed 30,000 community health workers in all communities.

Several researchers have reported that lack of access to improved sanitation forces the urban poor to use unhygienic pit latrines or polythene bags and/or discharge into nearby open storm drains and natural watercourses, creating severe environmental contamination and disease-related hazards [18-20]. Open defecation is common practice (37%) in rural areas of Ethiopia; it is also practiced by 8.2%, 5.8%, and 8.0% of slum residents in Addis Ababa, the total Addis Ababa population, and all urban areas of the country, respectively. The majority of Addis Ababa’s slum dwellers (88.6%) and 73% of its total population use unimproved sanitation facilities, showing that the urban poor are the population segment with the poorest access to sanitation services [6]. In conclusion, urban sanitation coverage is far from the MDG target and the majority of urban residents live with high health and environmental risks.

Most attention on monitoring sanitation growth worldwide has focused on household-level inventories (type and number of toilet infrastructures), ignoring proper utilization and user behavior [15]. The Ethiopian national inventories by different organizations such as MoH, JMP, and CSA lack data on utilization of improved sanitation technologies and user behavior, precluding a proper evaluation of the current state of access to improved sanitation. As indicated by Bartram and Cairncross [2], different levels of access along the sanitation ladder provide widely varying health benefits. For instance, the change from open defecation to the use of improvised latrines is a step forward but is unlikely to offer health benefits unless the latrine provides an adequate barrier between the users and their excreta. These incomparable sanitation coverage data resulted mainly from the absence of detailed guidelines and appropriate tools. Hence, in post-2015 MDGs, guidelines and tools should consider functions of sanitation systems in a closed-loop approach (using waste as a potential resource by both purifying and recycling) and examine user behavior in addition to using the hierarchy of predefined sanitation technologies as depicted in the sanitation ladder.

Dry pit latrines (both improved pit latrines and simple pit latrines), used by 92.5% of the Ethiopian population, require regular maintenance, particularly pit emptying and proper fecal sludge management (FSM). In the national inventory, pit levels, pit emptying practices, and FSM are not documented. FSM, the most important sanitation element, is also largely ignored in the global estimation of improved sanitation coverage. Baum et al. [12] indicated that estimating toilet facilities connected to sewage without treating and redefining them as unimproved sanitation reduced the estimates of improved sanitation coverage in 2010 by about 22%. Adequate treatment and valorization of fecal sludge have been absent in Ethiopia. As a result, none of the sanitation facilities in Ethiopia would qualify as improved sanitation facilities if the chain FSM system was included as a monitoring criterion. Hence, access to improved sanitation in the post-MDG era should also consider adequate treatment and valorization of fecal sludge as indicators of access to ISC.

### Trends in access to improved sanitation coverage

Although the trend of access to sanitation coverage in Ethiopia increased from 4% in 1990 to 47.9% in 2014, it falls short of the MDG target of 56%. Whereas the discrepancies in the trend analyses by the AGR and the JMP on one hand and the Ethiopian-CSA on the other can be explained methodologically, rapid population growth, high urbanization rates, and lack of political will to improve sanitation levels are the major drivers of low ISC in Ethiopia and apparently also in Sub-Saharan Africa overall. According to the trend analysis by Hopewell and Graham [21], in 31 major Sub-Saharan Africa cities, nearly half of them, including Addis Ababa, did not make progress in reducing open defecation from 2000 to 2012. The slow progress in increasing access to improved sanitation in Ethiopia and other developing countries can also be attributed to the lack of contextualized strategies, policies, and actions [22,23]; weak sectoral coordination; and low national budget allocation [24].

In addition to the observed differences in trends of ISC among the reports examined here, higher variability in ISC trends was observed in AGRs than in the JMP reports. Strong variability within the AGRs in sanitation coverage in Ethiopia was reported by Kumie and Ali [25]. Based on our experiences and observations, this variability appears to be associated with the absence of internal controls and audits that would ensure the reliability and integrity of reports related to sanitation coverage at each unit of administration in addition to the lack of standardized methods for gauging access to improved sanitation. Data routinely reported through government structures reflect only cumulative totals of facilities based on records from government-supported programs without follow-up monitoring to assess their utilization and maintenance.

Debates continue around the issue of how accessibility of improved sanitation is calculated, pointing out the need for standardized methods and protocols. For example, current estimation of access to improved sanitation worldwide, using type of latrine technology as an indicator, is inadequate [2,9] without considering the chain of the FSM system from containment to adequate treatment as well as proper utilization and user behavior. Only evaluation of these various components can provide adequate information on barriers between latrine users and their excreta.

Although household surveys are generally believed to provide the most accurate available data, all the appraised surveys cited in this manuscript lack a clear definition and boundary for the distinction between urban and rural. This lack is due in part to the difficulty of distinguishing between urban and rural communities in Ethiopia [26]. All the household surveys also fail to select representative samples from both urban and rural populations that consider socioeconomic and cultural attributes to distinguish different groups.

### Limitations

Access to improved sanitation in urban slums was studied only in the capital city of Addis Ababa, which may not be representative of the sanitation conditions of towns nationwide. The sanitation trend does not include annual variations since the surveys were conducted at several year intervals. The use of survey data collected from only one household member might bias results.

## Conclusion

Access to improved sanitation is a human right. On the road to universal access to improved sanitation for all, more than half of the Ethiopian population has no access to improved sanitation. In both urban and rural Ethiopia, access to improved sanitation coverage is far from the MDG target and the majority of residents are living with high health and environmental risks. The high proportion (88.6%) of Addis Ababa urban slum dwellers and of urban residents nationwide (82.5%) using unimproved sanitation facilities indicates that the urban poor have as low sanitation services coverage as the rural populations. Even this may underestimate actual coverage, which might be better gauged if the method of estimating improved sanitation coverage considered the functioning and utilization of sanitation systems and fecal sludge management (FSM) rather than simply identifying and counting available sanitation technologies. Lack of a standardized monitoring and reporting system has resulted in big disparities in sanitation trends among reports that use different monitoring methods. Dry pit latrines remain the most widely used toilet, accounting for about 97.5% of the improved sanitation coverage nationwide. However, their proper utilization and maintenance are not included as indicators for measuring access to improved sanitation coverage. The inadequate progress towards achieving the MDG target and the need to further expand sanitation coverage in the post-MDG era require urgent intensification of current intervention efforts and developing more coordinated actions. Review of policies and strategies is also required to improve planning, implementation, monitoring, and evaluation of sanitation interventions.

## Abbreviations

AGRs: Administrative government reports
CSA: Central statistical authority
FSM: Fecal sludge management
FT: Flush toilets
ISC: Improved sanitation coverage
ISL: Improved shared latrine
JMP: Joint monitoring program
MDG: Millennium development goal
MoFED: Ministry of finance & economic development
MoWIE: Ministry of water, irrigation & energy
NR: Not reported
OD: Open defecation
PIPL: Private improved pit latrine
PTPL: Private traditional pit latrine
UIS: Unimproved sanitation
UN: United Nations
UNICEF: United Nation Children’s Fund’s
USTs: Unsanitary toilets
WHO: World Health Organization

## References

[1] Prüss-Üstün A, Bos R, Gore F, Bartram J (2008). Safer water, better health: costs, benefits and sustainability of interventions to protect and promote health.

[2] Bartram J, Cairncross S (2010). Hygiene, sanitation, and water: forgotten foundations of health. PLoS Med.

[3] UN. General Assembly of the United Nations Resolutions 64th Session: Resolution No. A/RES/64/292 28 July 2010. Available at http://www.un.org/en/ga/64/.

[4] WHO. Drinking‐water, sanitation, and health. Resolution 64/24, UNWHAOR, 64th Session, UN Doc. A64/24; 2010. Available at http://apps.who.int/gb/ebwha/pdf_files/WHA64/A64_R24-en.pdf.

[5] WHO/UNICEF (2010). Joint monitoring programme for water supply and sanitation, progress on sanitation and drinking water: 2010 update.

[6] WHO/UNICEF (2014). Progress on drinking water and sanitation: 2014 update.

[7] CSA (2014). Ethiopia mini demographic and health survey 2014.

[8] MoFED (2012). Millenium development goals: Ethiopia MDGs report 2012.

[9] Mara D, Lane J, Scott B, Trouba D (2010). Sanitation and health. PLoS Med.

[10] Davis M (2006). Planet of slums. New Perspect Q.

[11] Moe CL, Rheingans RD (2006). Global challenges in water, sanitation and health. J Water Health.

[12] Baum R, Luh J, Bartram J (2013). Sanitation: a global estimate of sewerage connections without treatment and the resulting impact on MDG progress. Environ Sci Technol.

[13] Bostoen K, Evans B (2008). Crossfire: measures of sanitation coverage for the MDGs are unreliable, only raising a false sense of achievement’. Waterlines.

[14] Selendy JMH (2011). Water and sanitation-related diseases and the environment: challenges, interventions, and preventive measures.

[15] Kvarnström E, McConville J, Bracken P, Johansson M, Fogde M (2011). The sanitation ladder-a need for a revamp?. J Water, Sanitation Hygiene Dev.

[16] WHO, UNICEF (2008). Progress on drinking water and sanitation-special focus on sanitation.

[17] Galan DI, Kim SS, Graham JP (2013). Exploring changes in open defecation prevalence in sub-Saharan Africa based on national level indices. BMC Public Health.

[18] Isunju JB, Schwartz K, Schouten MA, Johnson WP, van Dijk MP (2011). Socio-economic aspects of improved sanitation in slums: a review. Public Health.

[19] Kwiringira J, Atekyereza P, Niwagaba C, Günther I (2014). Descending the sanitation ladder in urban Uganda: evidence from Kampala Slums. BMC Public Health.

[20] Konteh FH (2009). Urban sanitation and health in the developing world: reminiscing the nineteenth century industrial nations. Health Place.

[21] Hopewell MR, Graham JP (2014). Trends in access to water supply and sanitation in 31 major sub-Saharan African cities: an analysis of DHS data from 2000 to 2012. BMC Public Health.

[22] Mara D (2012). Sanitation: what’s the real problem?. IDS Bulletin.

[23] Cairncross S, Bartram J, Cumming O, Brocklehurst C (2010). Hygiene, sanitation, and water: what needs to be done?. PLoS Med.

[24] Kumie A, Ali A (2005). An overview of environmental health status in Ethiopia with particular emphasis to its organization, drinking water and sanitation: a literature survey. Ethiop J Health Dev.

[25] Sparkman D (2012). More than just counting toilets: the complexities of monitoring for sustainability in sanitation. Waterlines.

[26] Kloos H, Adugna A (1989). The Ethiopian population: growth and distribution. Geogr J.

